# Association of tet methylcytosine dioxygenase 2 and 5-hydroxymethylcytosine in endometrioid adenocarcinoma and its clinical significance

**DOI:** 10.1186/s12905-024-03021-2

**Published:** 2024-03-21

**Authors:** Lei Kuang, Jingbo Zhang, Yanyu Li, Qing Wang, Jianwei Liu, Bei Zhang

**Affiliations:** Department of Obstetrics and Gynecology, Xuzhou Central Hospital, Xuzhou Clinical School of Xuzhou Medical University, No. 199 South Jiefang Road, Xuzhou, 221009 China

**Keywords:** Endometrial cancer, Tet methylcytosine dioxygenase 2, 5-hydroxymethylcytosine, Association, Prognosis

## Abstract

**Background:**

Aberrant DNA methylation is a vital molecular alteration commonly detected in type I endometrial cancers (EC), and tet methylcytosine dioxygenase 2 (TET2) and 5-hydroxymethylcytosine (5hmC) play significant roles in DNA demethylation. However, little is known about the function and correlation of TET2 and 5hmC co-expressed in EC. This study intended to investigate the clinical significance of TET2 and 5hmC in EC.

**Methods:**

The levels of TET2 and 5hmC were detected in 326 endometrial tissues by immumohistochemistry, and the correlation of their level was detected by Pearson analysis. The association between the levels of TET2 and 5hmC and clinicopathologic characteristics was analyzed. Prognostic value of TET2 and 5hmC was explored by Kaplan–Meier analysis. The Cox proportional hazard regression model was used for univariate and multivariate analyses.

**Results:**

Based on the analysis results, TET2 protein level was positively correlated with 5hmC level in EC tissues (*r* = 0.801, *P* < 0.001). TET2^+^5hmC^+^ (high TET2 and high 5hmC) association was significantly associated with well differentiation, myometrial invasion, negative lymph node metastasis, and tumor stage in EC. Association of TET2 and 5hmC was confirmed as a prognostic factor (HR = 2.843, 95%CI = 1.226–3.605, *P* = 0.007) for EC patients, and EC patients with TET2^−^5hmC^−^ level had poor overall survival.

**Conclusions:**

In summary, the association of TET2 and 5hmC was downregulated in EC tissues, and may be a potential poor prognostic indicator for EC patients. Combined detection of TET2 and 5hmC may be valuable for the diagnosis and prognosis of EC.

## Introduction

Endometrial cancer (EC) is one of the most prevalent gynecological cancers, which accounts for 20%–30% of female genital malignancies and 7% of female systemic malignancies [1]. In recent years, the incidence of EC in China has increased year by year, and the age of onset is getting younger and younger [2]. Although early diagnosis results in a good overall prognosis for most EC patients, 15%-20% of EC patients still experience postoperative recurrence and tumor metastasis [3]. In addition, although the treatment plan of surgery combined with radiotherapy and chemotherapy can achieve good therapeutic effect, the risk of postoperative reproductive function loss and recurrence brought about by conservative treatment still poses a serious threat to the physical and mental health of patients with EC. The prognosis of advanced or recurrent EC is poor, and the 5-year survival rate is significantly reduced, about 17%-30% [4, 5]. Accordingly, it is of utmost importance to investigate the pathogenesis of EC, find new therapeutic targets, and explore effective prognostic indicators.

The development of EC is a multistep process involving many molecular biological changes. EC has been shown to be a complex disease driven by abnormal genetic and epigenetic alterations, as well as environmental factors. DNA methylation (generating 5-methylcytosine [5mC]) and hydroxymethylation (generating 5-hydroxymethylcytosine [5hmC]) are significant epigenetic modifications that are frequently found in cancer. Aberrant DNA methylation is a significant biochemical change frequently detected in EC. Ten-eleven translocation (TET) protein family, including TET1, 2 and 3, belongs to the α-ketoglutarate and ferrous ion-dependent dioxygenase family. All three proteins play a vital role in DNA demethylation [6], and can catalyze 5-methylcytosine (5-mC) to 5-hydroxymethylcytosine (5hmC) [7]. The dynamic balance of 5-mC and 5hmC is essential for epigenetic stability, and is involved in the development of multiple solid tumors as a common epigenetic modification [8, 9]. The TET2 gene, which is found in chromosome 4q24 and has 11 exons with a total length of 150 kb, encoding TET2 protein, and TET2 gene, which is produced after a gene fission event during vertebrate evolution, lacks the CpG DNA binding CXXC domain which exists in TET1 and TET3 [10]. Depletion of TET2 can cause DNA hypermethylation at up to 25% of active transcriptional enhancer elements in human hematopoietic cells [11]. Depletion of TET2 in mouse embryonic stem cells leads to significant loss of 5hmC at enhancers, along with an increase in enhancer hypermethylation and a decrease in enhancer activity [12]. Abnormal level of TET2 and 5hmC has been demonstrated in various cancers, such as leukemia [13, 14], breast cancer [15], colorectal cancer [16, 17], and melanoma [18]. However, the role of TET2 and 5hmC two in EC is poorly understood.

In another study, we compared TET2 level between different histopathological samples (Endometrioid endometrial adenocarcinoma, Serous endometrial adenocarcinoma, Mixed serous and endometrioid). The results showed that the level of TET2 was significantly different among different histopathological samples, and it was significantly over-expressed in endometrioid endometrial adenocarcinoma [19]. Endometrioid endometrial adenocarcinoma accounts for 80–90% of the pathological types of EC. In this study, the level of TET2 and 5hmC in EC and normal proliferative endometrium was evaluated by immunohistochemistry. Further, we analyzed the relationship between TET2 and 5hmC as well as with clinical characteristics and overall survival of EC patients. This study intended to examine the clinical significance of TET2 and 5hmC in EC.

## Methods

### Tissue samples and clinical data

This was a retrospective study. Endometrial samples were collected from 326 patients who had received surgery in the Department of Gynecological Oncology, Xuzhou Central Hospital (Xuzhou, China) from October 2012 and June 2014. This study included 62 normal endometrial (NE) tissues and 264 EC tissues. The NE tissues were extracted from women having hysterectomy for conditions such as uterine prolapse or fibroids. Clinical information was collected from medical record, including age, menopausal status, differentiation, myometrial invasion, lymph node metastasis, and clinical stage. Tumor staging of the EC samples was determined following the International Federation of Gynecology and Obstetrics (FIGO) criteria [20], and histological grade and type following World Health Organization classification [21].

Inclusion criteria were as follows: (1) a diagnosis of endometrial adenocarcinoma or normal proliferative endometrial tissue confirmed by pathological examination; (2) complete clinical information; and (3) outpatient or telephone follow-up after treatment. Exclusion criteria were as follows: (1) preoperative treatment with radiotherapy, chemotherapy, or targeted drugs; (2) presence of other systemic malignancies, or metastatic carcinoma from other organ tumors of the reproductive system (including multiple primary cancer); (3) a history of out-of-hospital treatment.

All 264 patients with EC received outpatient or telephone follow-up. The beginning of treatment was the start of follow-up, and June 30, 2019 was the end of follow-up end. Patients who died from an accident or other unrelated illness were reported as censored events. This study was approved by the Ethics Committee of Xuzhou Central Hospital and each patient signed the informed consent.

### Immunohistochemistry (IHC) analysis

Paraffin-embedded tissue blocks were serially cut at a thickness of 4 μm. The obtained sections were treated with 3% hydrogen peroxide, and antigen retrieval was performed in a high-temperature environment. Next the sections were incubated at 4 °C overnight with primary antibodies TET2 (ab94580, Abcam, UK) and 5hmC (ab214728, Abcam, UK) at 1:200 dilution, and then with peroxidase-labeled secondary antibody (Sigma–Aldrich) for 30 min at 37 °C. 3,3′-Diaminobenzidine (Affinity, ZLI-9018) substrate was responsible for color development. Instead of the primary antibody, phosphate buffered saline (Sigma–Aldrich) was utilized as a negative control. A double-blind analysis of the sections was performed by two experienced pathologists. TET2 positivity was visible as yellow–brown granules in the nucleus and cytoplasm, and 5hmC positive staining was visible as a brownish yellow color in the nucleus. The intensity of IHC staining for cells in endometrial tissue was rated as 0 (negative), 1 (weakly positive), 2 (moderately positive), and 3 (strongly positive). The final staining score of tissue = (1 × percentage of weakly positive cells + 2 × percentage of moderately positive cells + 3 × percentage of strongly positive cells) × 100, and the final staining score ranged from 0 to 300. Using X⁃Tile software, the level of TET2 and 5hmC was classified into “low or no” level and “high” level based on the survival prognosis of EC patients. The cut-off value for TET2 and 5hmC was selected as 130. Tissues with staining score 0–130 were deemed as “low or no level”, which is denoted by “ ^−^”, while 131–300 was regarded as “high level”, which is denoted by “ ^+^”.

### Statistical analysis

Statistical analysis was carried out using SPSS 22.0 statistical software (SPSS Inc., USA). The association between clinicopathologic characteristics and TET2/5hmC level was examined using chi-square test. Pearson correlation analysis was conducted to find the link between TET2 and 5hmC in EC tissues. The Kaplan–Meier method was utilized for the survival analysis, and the log-rank test for comparison of differences in the overall survival rate. Both univariate and multivariate analyses were conducted using the Cox proportional hazard regression model. Statistical significance was defined as *P* < 0.05.

## Results

### Level of TET2 and 5hmC in endometrial tissues

**Fig. 1:**
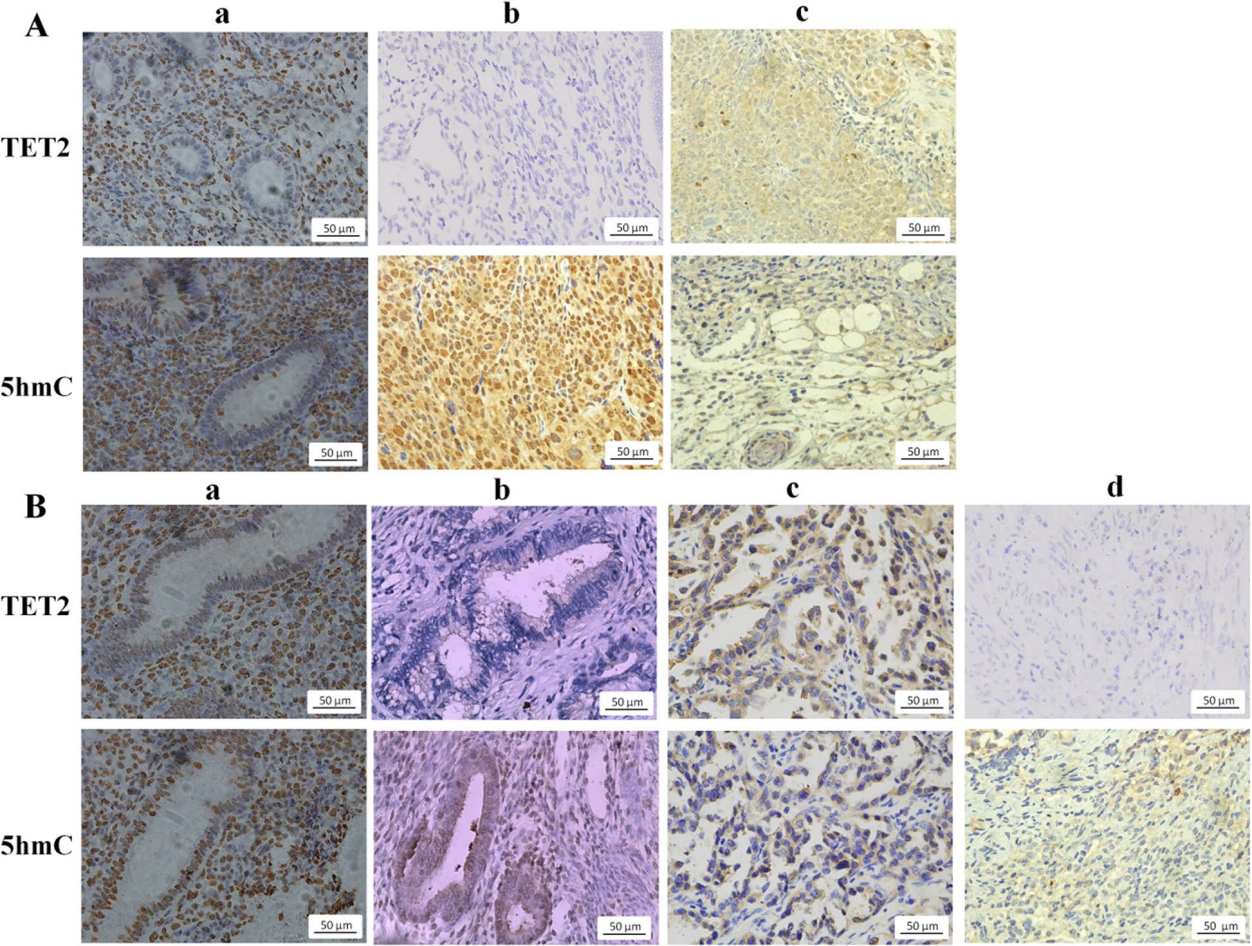
Representative staining images of TET2 and 5hmC in endometrial tissues. **A** Normal endometrial tissues with the levels of TET2 and 5hmC. (a) High level of TET2 and 5hmC; (b) Low level of TET2 and high level of 5hmC; (c) High level of TET2 and low level of 5hmC. (d) Low level of TET2 and 5hmC. **B** Endometrial cancer tissues with the level of TET2 and 5hmC. (a) High level of TET2 and 5hmC; (b) Low level of TET2 and high level of 5hmC; (c) High level of TET2 and low level of 5hmC. (d) Low level of TET2 and 5hmC

As shown in Fig. 1A–B, TET2-positive staining appeared as yellow–brown granules in the nucleus and cytoplasm, while 5hmC-positive staining as brownish yellow in the nucleus. According to the staining score, endometrial tissues were classified as high TET2 and high 5hmC (TET2^+^5hmC^+^), high TET2 and low 5hmC (TET2^+^5hmC^−^), low TET2 and high 5hmC (TET2^−^5hmC^+^), and low TET2 and low 5hmC (TET2^−^5hmC^−^). The rate of TET2^+^5hmC^+^, TET2^+^5hmC^−^, TET2^−^5hmC^+^, and TET2^−^5hmC^−^ in EC tissues were 51.52%, 0.76%, 7.95%, and 38.26% respectively. EC tissues had a lower rate of TET2^+^5hmC^+^ than that in NE tissues (51.52% *vs.* 79.03%), and TET2^−^5hmC^−^ was not detected in normal endometrium (Table 1).

**Table 1:** Association of TET2 and 5hmC in endometrial tissues

Characteristic	n	TET2	+	+	-	-	χ^2^	*P*
		5hmC	+	-	+	-		
Normal proliferative endometrium	62		49 (79.03)	5 (8.06)	8 (12.90)	0 (0.00)	36.793	< 0.001*
Endometrioid endometrial adenocarcinoma	264		136 (51.52)	6 (0.76)	21 (7.95)	101 (38.26)		

+ : high level, -: Low or no level, ^*^P <0.05

### Correlation of 5hmC and TET2 in EC tissues

**Fig. 2:**
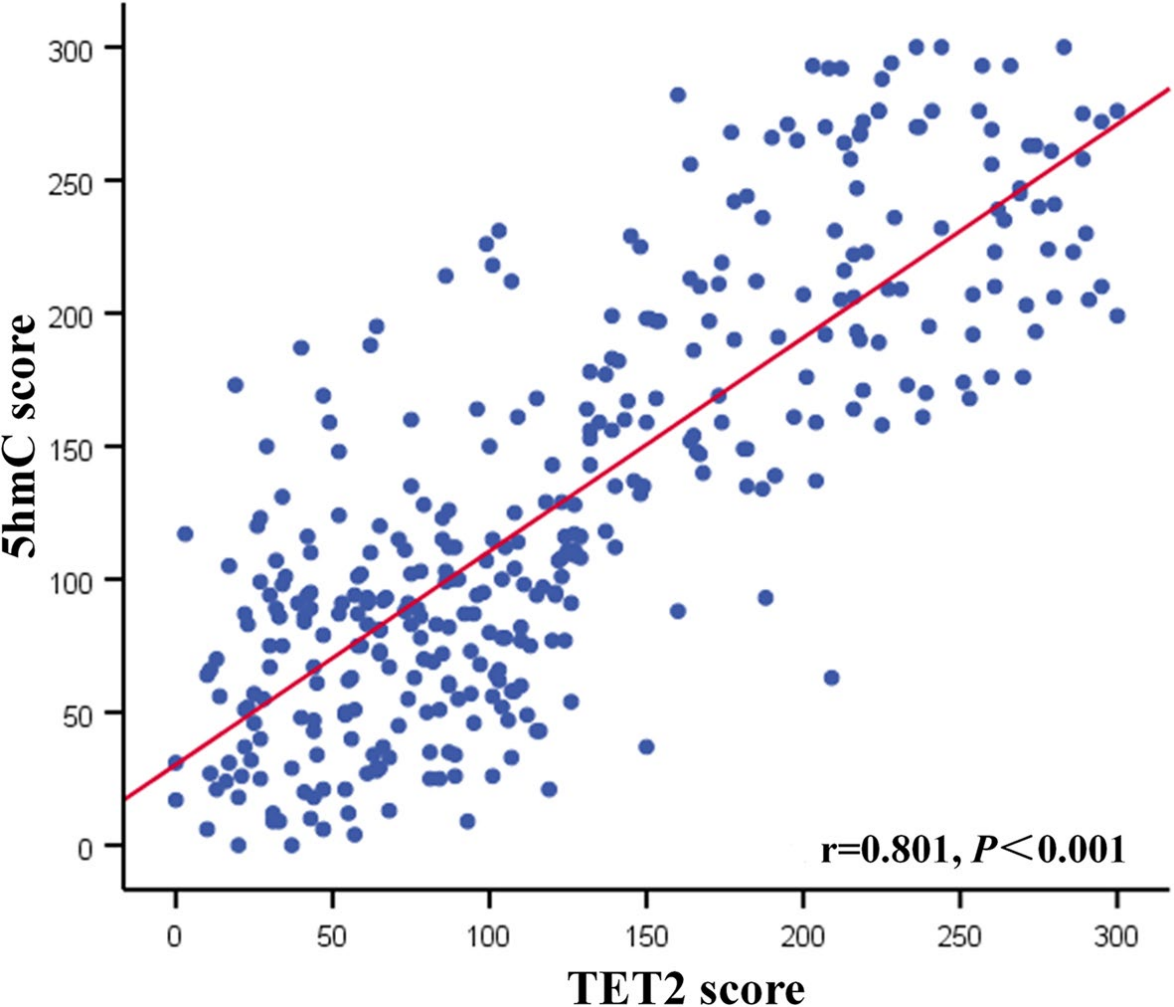
Correlation analysis of TET2 and 5hmC in endometrial cancer tissues

The relationship between TET2 and 5hmC was discovered using a Pearson correlation analysis. As shown in Fig. 2, TET2 level was positively correlated with 5hmC (*r* = 0.801 and *P* < 0.001).

### Relationship between clinicopathologic characteristics and TET2/5hmC association in EC

The relationship between TET2/5hmC association and clinicopathologic characteristics was explored in 264 EC patients (Table 2). The results showed that TET2/5hmC association was significantly associated with differentiation (χ^2^ = 82.249, *P* < 0.001), myometrial invasion (χ^2^ = 44.060, *P* < 0.001), lymph node metastasis (χ^2^ = 15.231, *P* = 0.002), and FIGO stage (χ^2^ = 38.769, *P* < 0.001). However, there was no correlation between TET2/5hmC association and age or menopausal status (*P* > 0.05).

**Table 2:** Association of TET2 and 5hmC and clinicopathological characteristics of EC patients

Characteristics	n	TET2	+	+	-	-	χ^2^	*P*
		5hmC	+	-	+	-		
Total	264		136 (51.52)	6 (0.76)	21 (7.95)	101 (38.26)		
Age (year)							4.857	0.183
≥ 45	225		121 (53.78)	4 (1.78)	16 (7.11)	84 (37.33)		
< 45	39		15 (38.46)	2 (5.13)	5 (12.82)	17 (43.59)		
Menopausal status							2.077	0.557
Pre-menopause	93		52 (55.91)	3 (3.23)	6 (6.45)	32 (34.41)		
Post-menopause	171		84 (49.12)	3 (1.75)	15 (8.77)	69 (40.35)		
Differentiation							82.249	< 0.001*
Well	166		119 (71.69)	3 (3.61)	12 (7.23)	32 (19.28)		
Moderate	37		10 (27.03)	0 (0.00)	3 (8.11)	24 (64.86)		
Poor	61		7 (11.48)	3 (4.92)	6 (9.84)	45 (73.77)		
Myometrial invasion							44.060	< 0.001*
< 1/2	174		112 (64.38)	5 (2.87)	15 (8.62)	42 (24.14)		
≥ 1/2	90		24 (26.67)	1 (1.11)	6 (6.67)	59 (65.56)		
Lymph node metastasis							15.231	0.002*
Negative	240		132 (55.00)	4 (1.67)	18 (7.50)	86 (35.83)		
Positive	24		4 (16.67)	2 (8.33)	3 (12.50)	15 (62.50)		
FIGO Stage							38.769	< 0.001*
I	195		121 (62.05)	3 (1.54)	15 (7.69)	56 (28.72)		
II	24		6 (25.00)	2 (8.33)	2 (8.33)	14 (58.33)		
III	45		9 (20.00)	1 (2.22)	4 (8.89)	31 (68.89)		

*P <0.05

### Correlation of TET2/5hmC association with prognosis of EC patients

Prognostic variables for EC patients were identified using both univariate and multivariate analysis. Univariate analysis showed that TET2/5hmC association (*P* < 0.001), differentiation (*P* = 0.036), lymph node metastasis (*P* = 0.015), and FIGO stage (*P* = 0.006) were significantly related to overall survival of EC patients. Multivariate analysis further demonstrated TET2/5hmC association (HR: 2.843; 95%CI: 1.226–3.605; *P* = 0.007), lymph node metastasis (HR: 5.241; 95%CI: 1.169–13.856; *P* = 0.037), and FIGO stage (HR: 3.979; 95%CI: 2.153–6.780; *P* = 0.023) were independent prognostic factors for EC patients (Table 3).

**Table 3:** Univariate and multivariate analyses of the prognostic factors for overall survival in EC

	Univariate analysis			Multivariate analysis		
	HR	*P* value	95%CI	HR	*P* value	95%CI
TET2 and 5hmC level						
T^+^5^+^ vs T^+^5^−^vs T^−^5^+^ vs T^−^5^−^	3.035	< 0.001*	1.751–5.259	2.843	0.007*	1.226–3.605
Age (year)						
	0.989	0.986	0.308–3.181			
Menopausal status						
Pre-vs Post-menopause	1.056	0.835	0.634–1.759			
Differentiation						
Well vs Moderate vs Poor	1.812	0.036*	1.040–3.157			
Myometrial invasion						
< 1/2 vs ≥ 1/2	0.955	0.868	0.551–1.653			
Lymph node metastasis						
Negative vs Positive	5.828	0.015*	1.329–14.615	5.241	0.037*	1.169–13.856
FIGO Stage						
I vs II vs III	4.261	0.006*	2.347–7.154	3.979	0.023*	2.153–6.780

*HR* Hazard ratio, *CI* Confidence interval

^*^P < 0.05

**Fig. 3:**
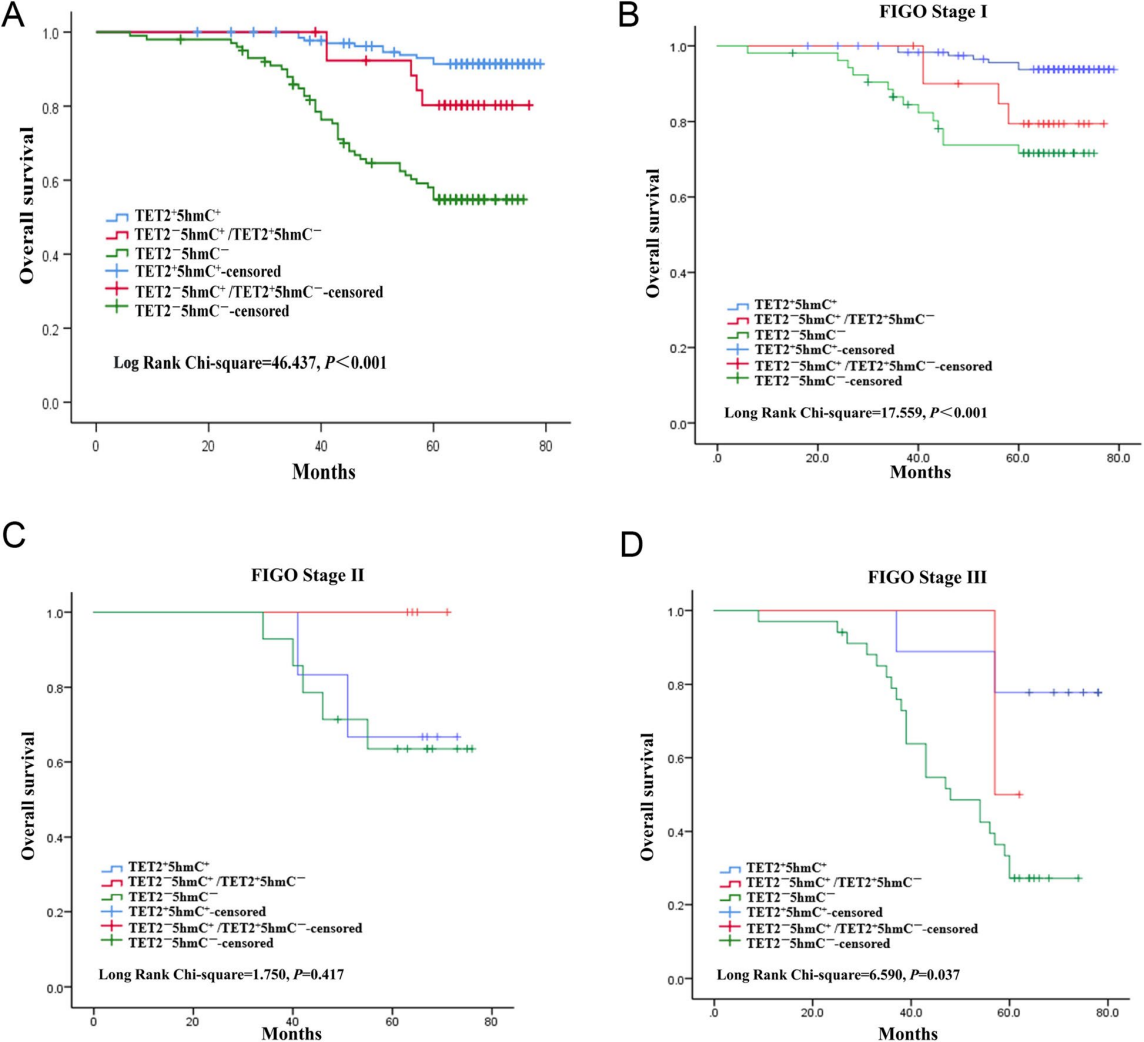
Survival curve of endometrial cancer patients using the Kaplan–Meier method and the log-rank test. **A** Overall survival curves for patients with TET2^+^5hmC^+^(blue line), TET2^−^5hmC^+^ /TET2^+^5hmC^−^ (red line) and TET2^−^5hmC^−^(green line). **B**-**D**, Analysis of the association between TET2^+^5hmC^+^(blue line), TET2^−^5hmC^+^ /TET2^+^5hmC^−^ (red line) and TET2^−^5hmC^−^(green line) level and poor overall survival in patients with FIGO Stage I, Stage II, and Stage III

Kaplan–Meier survival curve demonstrated that the overall survival rate of TET2 + 5hmC + EC patients was higher than that of TET2 + 5hmC + /TET2 + 5HMC-EC patients, and the overall survival time of TET2 + 5hmC + EC patients in FIGO stage I was significantly longer. Compared with TET2 + 5hmC + and TET2 + 5hmC + /TET2 + 5hmC-, patients with TET2-5HMC-EC had significantly lower overall survival rates and the lowest overall survival rates at all FIGO stages (Fig. 3). Hazard ratios were further calculated for disease stage, and the results showed an association between TET2^−^5hmC^−^ level and poor overall survival in FIGO Stage I (χ^2^ = 17.559, *P* < 0.001) and Stage III (χ^2^ = 6.590, *P* = 0.037) patients. There was no significant difference between TET2^−^5hmC^−^ level and adverse overall survival in patients with Stage II (χ^2^ = 1.750, *P* = 0.417) (Fig. 3B-D).

## Discussion

DNA methylation, an important epigenetic modification, controls gene expression and maintains genome structure [22]. Abnormal DNA methylation is frequently occurs in the promoter region of transcription factors. Tumorigenesis typically results from either hyper-or hypomethylation. DNA hypermethylation in tumor suppressor motifs leads to transcription inhibition and downregulation of tumor suppressor genes. DNA hypomethylation activates proto-oncogenes and affects chromosome stability [23]. DNA methylation is a characterization and reflection of early molecular variations in human tumors, indicating that the studies on DNA methylation is anticipated to provide novel methods for early identification and prevention of tumors.

Previous studies have found that both the TET protein family and 5hmC play important roles in tumor development and progression [24]. TET2 expression was lower in normal tissues than that in some epithelial neoplasms, such as breast adenocarcinoma and gastric cancer [25, 26]. However, Yang et al. confirmed that TET2 mRNA and protein level in hepatic cancerous tissues were both raised compared with normal hepatic tissues [27].

5-mC can be hydroxylated to 5hmC under the catalysis of TET proteins. 5hmC plays a key role in neural development, and abnormal 5hmC level was found in some neurological diseases such as Alzheimer's disease [28]. Mounting evidence has demonstrated that 5hmC is involved in the pathogenesis of various tumors. 5hmC level in some malignant tumors, like lung cancer, liver cancer, breast cancer and melanoma, was significantly decreased compared with normal tissues, whereas its level was increased in benign uterine leiomyoma [29–32]. In another study, we compared the differential level of TET2 between normal endometrial tissue and endometrial cancer tissue was observed using the endometrial cancer data in TCGA database [19]. The results showed that the level of TET2 was significantly different among different histopathological samples, and it was significantly over-expressed in endometrioid endometrial adenocarcinoma.

This study detected the association of 5hmC and TET2 in EC tissues. In comparison to normal endometrial tissues, EC tissues showed a lower rate of TET2^+^5hmC^+^ association (51.52% *vs.* 79.03%) and a significantly higher rate of TET2^−^/5hmC^−^ association (38.26% *vs.* 0.00%). Furthermore, TET2 level level was positively associated with 5hmC level in EC tissues, which was basically consistent with the result of Ciesielski et al. [33]. The association of TET2 and 5hmC was related to well differentiation, myometrial invasion, FIGO stage, and negative lymph node metastasis. These results suggested that high level of TET2 and 5hmC may inhibit EC progression.

Wang et al. stated that TET2 mutation was an independent prognostic factor for acute myeloid leukemia [34]. Downregulation of TET2 mRNA in prostate cancer was found to be strongly associated with reduced survival [35]. Similarly, 5hmC loss was linked to poor overall survival in ovarian cancer and pediatric central nervous system tumor [36, 37]. In this study, univariate analysis showed that the association of TET2 and 5hmC, differentiation, FIGO stage, and lymph node status were correlated with survival of EC patients. Further multivariate analysis revealed the association of TET2 and 5hmC, FIGO stage, and lymph node metastasis were independent factors affecting EC prognosis. Additionally, Kaplan–Meier survival curve proved that the association of TET2^−^5hmC^−^ predicted poor overall survival of EC patients. Collectively, association of TET2 and 5hmC is closely related to the prognosis of EC patients.

### Limitations

Our study suggests that abnormal level of TET2 and 5hmC may be a potential diagnostic and therapeutic target for EC. Thus the simultaneous detection of TET2 and 5hmC in clinical practice is of great significance for diagnosing and predicting the prognosis of EC. However, this study also has the limitation of a small sample size, which limits their clinical development and application. Therefore, our conclusion needs to be further validated by large-scale, multi-stage, and multi-center clinical trials.

## Conclusion

Our study suggests that abnormal level of TET2 and 5hmC may be a potential diagnostic and therapeutic target for EC. Thus the simultaneous detection of TET2 and 5hmC in clinical practice is of great significance for diagnosing and predicting the prognosis of EC. However, this study also has the limitation of a small sample size, which limits their clinical development and application. Therefore, our conclusion needs to be further validated by large-scale, multi-stage, and multi-center clinical trials.

## Data Availability

The dataset generated and/or analysed during the study are available from the corresponding author on reasonable request.

## Abbreviations

EC: Endometrial cancer
TET2: Tet methylcytosine dioxygenase 2
5hmC: 5-Hydroxymethylcytosine
5mC: 5-Methylcytosine
